# Encouraging Early Outcomes of Treatment With Arsenic Trioxide Combined With Chemotherapy for Alveolar Rhabdomyosarcoma in Children: 4 Case Reports

**DOI:** 10.3389/fonc.2021.751623

**Published:** 2021-10-29

**Authors:** Xiaomin Peng, Xilin Xiong, Yang Li, Chuchu Feng, Hongyan Liu, Pingping Wu, Chunmou Li, Wenjun Weng

**Affiliations:** ^1^ Department of Pediatric Hematology/Oncology, Sun Yat-Sen Memorial Hospital, Sun Yat-Sen University, Guangzhou, China; ^2^ Hematology and Oncology Department ward 7, Beijing Jingdu Children’s Hospital, Beijing, China

**Keywords:** alveolar rhabdomyosarcoma, arsenic trioxide, chemotherapy, children, ATO

## Abstract

**Background:**

Alveolar rhabdomyosarcoma (ARMS) is a subtype of rhabdomyosarcoma characterized by its aggressive behavior and poor prognosis, highlighting the need for novel treatment options. Arsenic trioxide (ATO) has been shown to specifically inhibit tumor growth and the metastasis of ARMS *in vitro* by acting on the hedgehog pathway. Here we report on a pilot clinical study to evaluate the activity of an ATO-combined chemotherapy approach for the treatment of ARMS patients.

**Methods:**

We designed a therapeutic schedule of an ATO-combined chemotherapy, incorporating comprehensive management according to the Intergroup Rhabdomyosarcoma Study Group protocol. ATO was administered at 0.16 mg/kg per day over 8 h *via* an IV for 10 days combined with a chemotherapeutic regimen of vincristine, actinomycin, and cyclophosphamide (VAC regimen) on the third day, which was repeated every 21 days. A total of eight cycles of ATO-combined chemotherapy were applied throughout the entire scheme.

**Results:**

A total of three refractory/recurrent and one untreated ARMS patient, three male and one female, with a median age of 5.8 years (range, 5.1 to 12.5 years), were enrolled in the present study. All patients were sensitive to combined chemotherapy with ATO and achieved partial or complete remission during treatment. Except for reversible gastrointestinal reaction and myelosuppression, no other adverse events were observed during the process of treatment.

**Conclusions:**

The combined chemotherapy of ATO and the VAC regimen exhibited beneficial activities against ARMS in pediatrics and was well tolerated, but prospective large-scale clinical trials are warranted to determine the long-term efficacy, optimal courses, and late toxicity in this population.

## Introduction

Rhabdomyosarcoma (RMS), the most common soft tissue sarcoma in pediatrics, is a highly malignant mesenchymal tumor considered to originate from immature striated muscles. RMS can present as a heterogeneous array of differing histological, genetic, and clinical features, and the most recent World Health Organization classification scheme subdivides RMS into four subtypes: embryonal RMS, alveolar RMS (ARMS), pleomorphic RMS, and spindle cell/sclerosing RMS (1). ARMS accounts for 20–30% of RMS cases and is more common in older children and young adults, often involving the trunk and extremities (1). ARMS is characterized by the presence of poorly differentiated rhabdomyoblasts in the histopathological analysis and shows a more aggressive behavior following the onset of metastasis or relapse. Patients with ARMS have a poor prognosis, with a 5-year survival of <50% (2).

The management of ARMS is facing a bottleneck, as chemotherapy still plays a critical role in the treatment of patients with ARMS. Presently, a combination of vincristine (VCR), actinomycin, and cyclophosphamide (VAC) remains the gold-standard protocol for ARMS therapy since few candidate drugs have shown superior durable objective response rates in clinical trials over the last few decades (3, 4). Recent molecular and genetic studies have produced novel insights into the biology and tumorigenesis of ARMS, which may imply novel therapies. It is well established that 70–80% of ARMS cases harbor chromosomal translocations t(2;13)(q35;q14) or t(1;13)(p36;q14), which generate paired box (PAX)3-forkhead box O1 (FOXO1) and PAX7-FOXO1 fusions, respectively. However, at present, there are no direct inhibitors against PAX-FOXO1 fusion oncoproteins, and targeting epigenetic cofactors is largely limited to preclinical trials.

Recent evidence has shown that the abnormal activation of the hedgehog (Hh) signaling pathway is prevalent in ARMS tumors (5–7). Arsenic trioxide (ATO), a Food and Drug Administration (FDA)-approved drug, was discovered through advanced research methods and was shown to be clinically useful against tumors exhibiting activated Hh signaling. Several *in vitro* experiments have confirmed the anti-tumor effects of ATO in inhibiting tumor growth and the invasion of ARMS cell lines (8, 9). Furthermore, another *in vitro* study identified the synergistic induction of apoptosis by ATO together with several anti-microtubule agents, including VCR (10). VCR is known as cell cycle phase M-specific anti-tubulin agent. ATO was reported to induce arrest in the G1 or G2/M phases of the cell cycle in most solid tumor cells. Thus, the pre-application of ATO followed by VCR may potentiate anti-tumor effects, as demonstrated in our previous study in neuroblastoma cell lines (11). More specifically, the IC_50_ of ATO on ARMS cell lines is 1.3–2.0 μM (9), which is very close to the effective serum concentration in the clinical treatment of acute promyelocytic leukemia (APL) (12). This suggests that similar dosage and administration of ATO in APL, given that the commonly used dose of ATO in APL is 0.16 mg/kg day (13, 14), could be effective in the treatment of ARMS. Based on the abovementioned information, an innovative combined therapy of ATO and VAC regimen was designed. Here we first report on four patients with ARMS who received ATO therapy combined with a VAC regimen and showed beneficial responses with good tolerance to date.

## Patients and Methods

**Figure 1:**
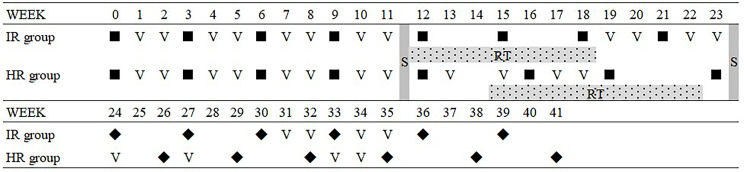
Comprehensive management protocol with the ATO-combined chemotherapy. ▪, ATO-combined chemotherapy with VAC regimen; ◆, VAC regimen; S, surgrey; RT, radiotherapy; ATO, arsenic trioxide; VAC, vincristine, actinomycin, and cyclophosphamide.

This was a case series pilot phase 2 clinical study. The eligible participants were ARMS patients under 18 years old without a major organ dysfunction. All patients received eight cycles of the ATO-combined chemotherapy incorporating comprehensive management according to the Intergroup Rhabdomyosarcoma Study Group protocol (**Figure 1**). The ATO injection was manufactured by Harbin Yida Pharmaceutical Co. Ltd.(Harbin, China). A total of 0.16 mg/kg per day of ATO was administered over 8 h by IV daily for 10 days with a combined chemotherapy of VAC regimen on the third day, which was repeated every 21 days (**Table 1**). After four cycles, radical surgery and/or radiotherapy was implemented for local control. All the parents of the patients involved in this study provided informed consent prior to study enrollment.

**Table 1:** Dose and usage of the arsenic trioxide-combined chemotherapeutics.

Drug	Dosage				Route of administration	Time
	≥3 years old	1-3 years old	<1 years old	Max		
Vincristine	1.5 mg/m^2^	0.05 mg/kg	0.025 mg/kg	2 mg	IV	Day 3
Actinomycin D	0.045 mg/kg	0.045 mg/kg	0.025 mg/kg	2.5 mg	IV-drip	Day 3
Cyclophosphamide	2.2 g/m^2^	73 mg/kg	36 mg/kg	–	IV-drip	Day 3
Arsenic trioxide	0.16 mg/kg day	0.16 mg/kg day	0.16 mg/kg day	–	IV-drip, PI > 8h	Days 1-10

Therapeutic response was evaluated at 4 weeks after the eight cycles of ATO-combined chemotherapy using the Response Evaluation Criteria in Solid Tumors, version 1.1. Adverse events were monitored and graded by the National Cancer Institute Common Terminology Criteria for Adverse Events, version 5.0.

## Results

**Figure 2:**
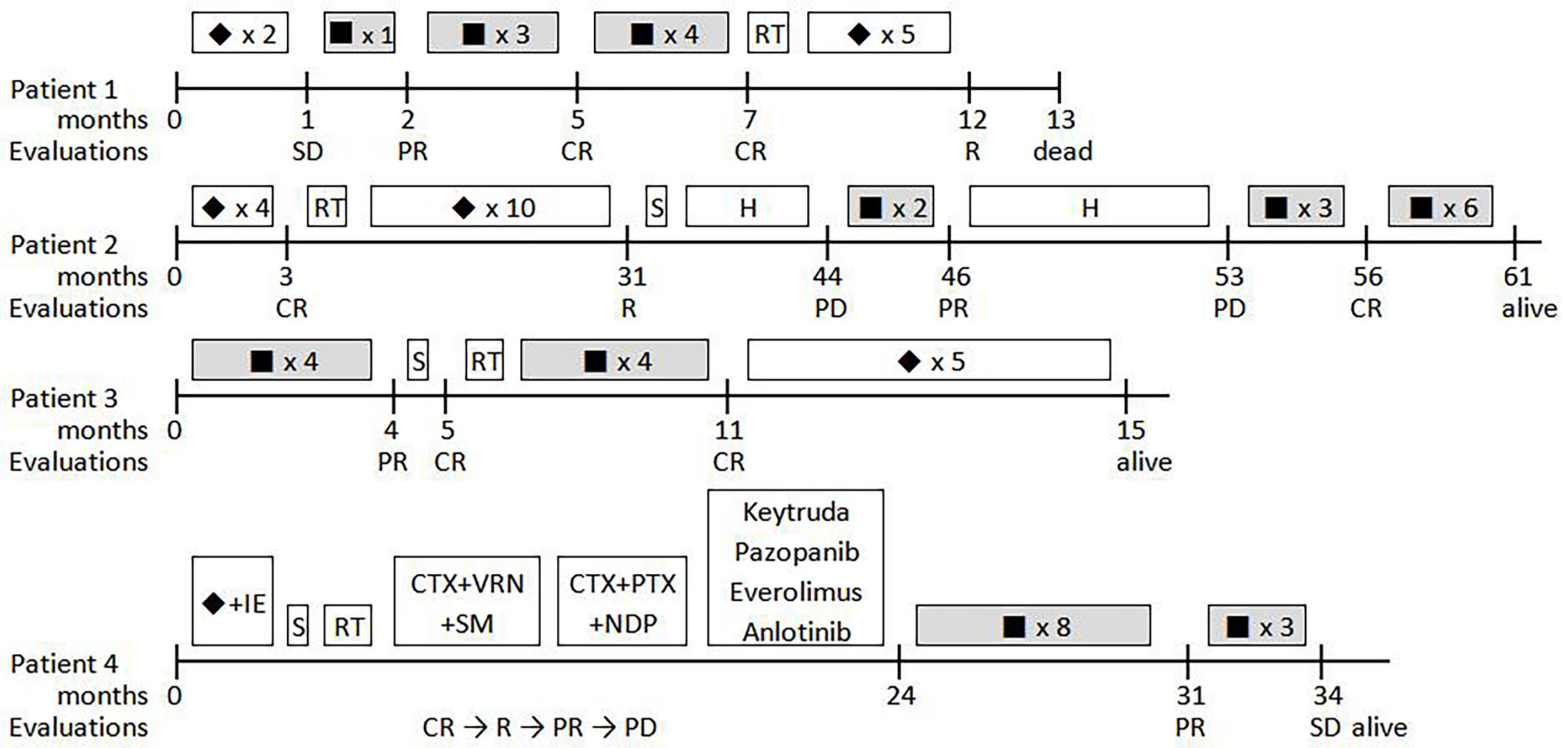
Timelines for the four patients treated with ATO-combined chemotherapy.▪, ATO-combined chemotherapy with VAC regimen; ◆, VAC regimen; S, surgery; RT, radiotherapy; H, herbs; CTX, cyclophosphamide; I, ifosfamide; E, etoposide; VRN, vinorelbine; SM, sirolimus; PTX, paclitaxel; NDP, nedaplatin; CR, complete remission; PR, partial remission; PD, progressive disease; SD, stable disease.

In total, four ARMS patients, three male and one female, with a median age of 5.8 years (range, 5.1 to 12.5 years), were enrolled in this study. The patient characteristics are summarized in **Table 2**, and the timelines for each case are depicted in **Figure 2**.

**Table 2:** Characteristics of the four patients with alveolar rhabdomyosarcoma treated with the ATO-combined chemotherapy regimen.

Patient	Gender	Age	Primary lesions	Tumor–node–metastasis stage	IRS group	Molecular finding	Status of disease	Previous treatment	Response to ATO	Toxicity	Outcome
1	M	5.1 years	Lower extremity	T2aN1M1	IV	PAX3-FOXO1 fusion-positive	Refractory	VAC	CR	Myelosuppression, vomiting	Died of recurrence
2	M	5.8 years	Maxillofacial region	T2bNx M0	IIIb	GLI1 amplification	Recurrent	VAC, V, RT	CR	Myelosuppression, vomiting	Survived and remained disease-free
3	M	12.5 years	Upper extremity	T1bN0M0	IIIa	PAX3-FOXO1 fusion-positive	Untreated	–	CR	Myelosuppression, vomiting	Survived and remained disease-free
4	F	4.3 years	Left buttock	T2bNx M1	IV	GLI2 amplification	Refractory	VAC, IE, CTX+VRN+SM, CTX+PTX+NDP, keytruda, pazopanib, everolimus anlotinib, RT	PR	Myelosuppression, vomiting	Survived with tumor

ATO, arsenic trioxide; V, vincristine; A, actinomycin D; C(CTX), cyclophosphamide; I, ifosfamide; E, etoposide; VRN, vinorelbine; SM, sirolimus; PTX, paclitaxel; NDP, nedaplatin; RT, radiotherapy; M, male; F, female; CR, complete remission; PR, partial remission; GLI, glioma-associated transcription factor; IRS, Intergroup Rhabdomyosarcoma Study.

Patient 1 was a 5-year-old, male child who presented with a mass in the right inguinal and popliteal fossa and marked edema of the homolateral leg. The pathological diagnosis of ARMS with PAX-FOXO1 fusion-positive was defined using an excision biopsy of the right inguinal mass, while a large number of tumor cells were found in the bone marrow examination. A positron emission tomography–computed tomography (PET–CT) scan showed a large mass in the mediastinum and the right lower extremity, and distant metastases were found in the parietal pleura, pancreas, multiple bone lesions, celiac, and iliac lymph nodes. Chemotherapy was started with two courses of the VAC regimen, but this failed to alleviate oncothlipsis, with a <30% decrease in the sum of the diameters of the tumor lesions. Then, ATO was added in the third course of the VAC regimen. Notably, the edema disappeared soon after, and the superficial masses shrank by 83% within 3 weeks. Meanwhile, residual disease in the bone marrow converted negative. After four courses of the ATO-combined chemotherapy, tumor response was evaluated *via* PET–CT, and the bone marrow examination suggested complete remission (CR). A total of eight courses of ATO treatment were performed, and the patient remained in CR for >7 months. By the end of a subsequent therapy without ATO, new tumor nodules in the brain were identified by PET–CT and magnetic resonance imaging (MRI), and the patient died after giving up treatment. The overall survival was 10.3 months from the beginning of ATO treatment.

**Figure 3:**
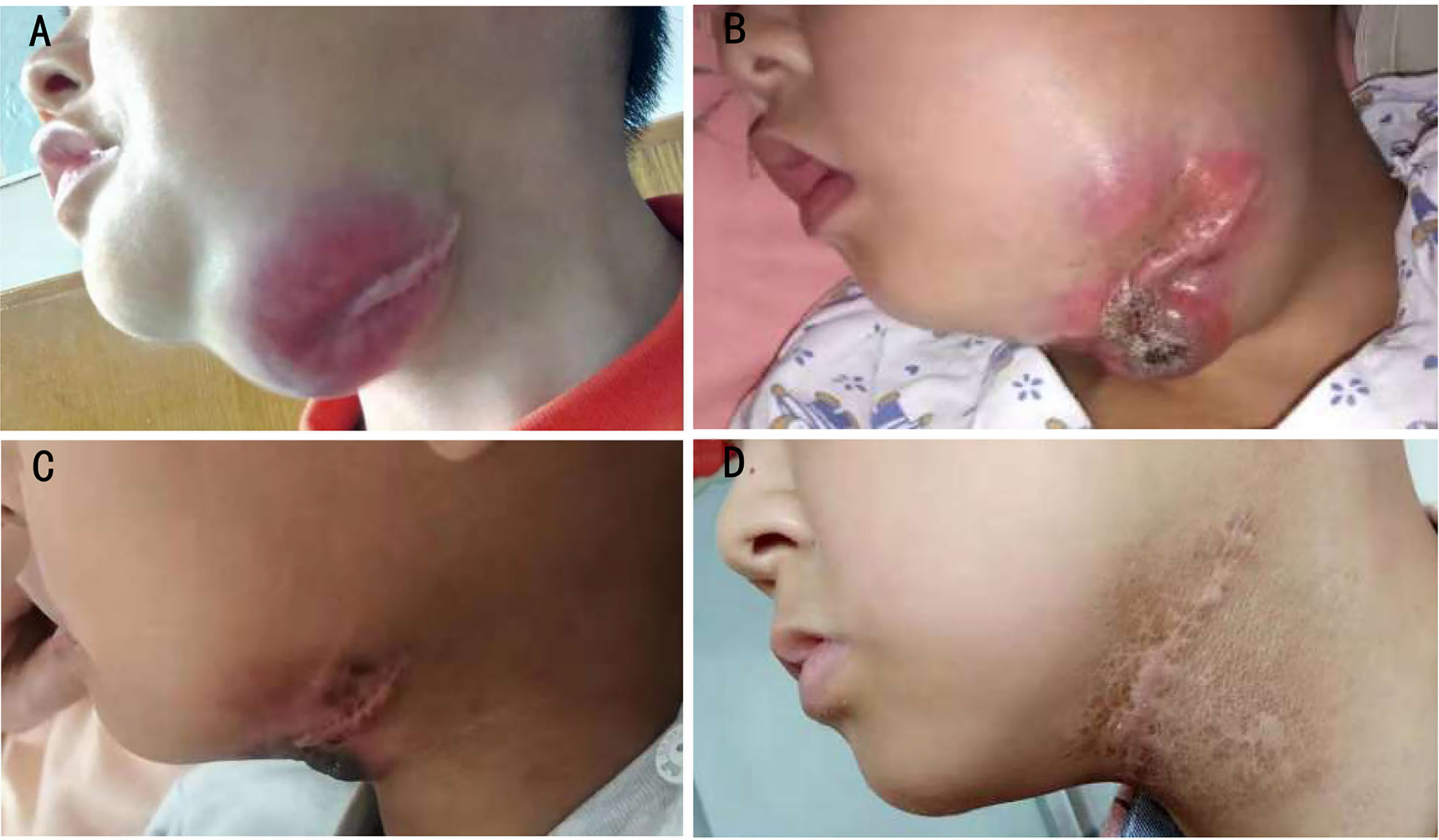
Changes in macroscopic tumor in patient 2. **(A)** Before ATO treatment, **(B)** after 7 months of treatment interruption, including two courses of the ATO-combined chemotherapy, **(C)** 2 days after two courses of treatment with the ATO-combined chemotherapy (four courses in total), and **(D)** 3 months after seven courses of treatment with the ATO-combined chemotherapy (nine courses in total). ATO, arsenic trioxide.

**Figure 4:**
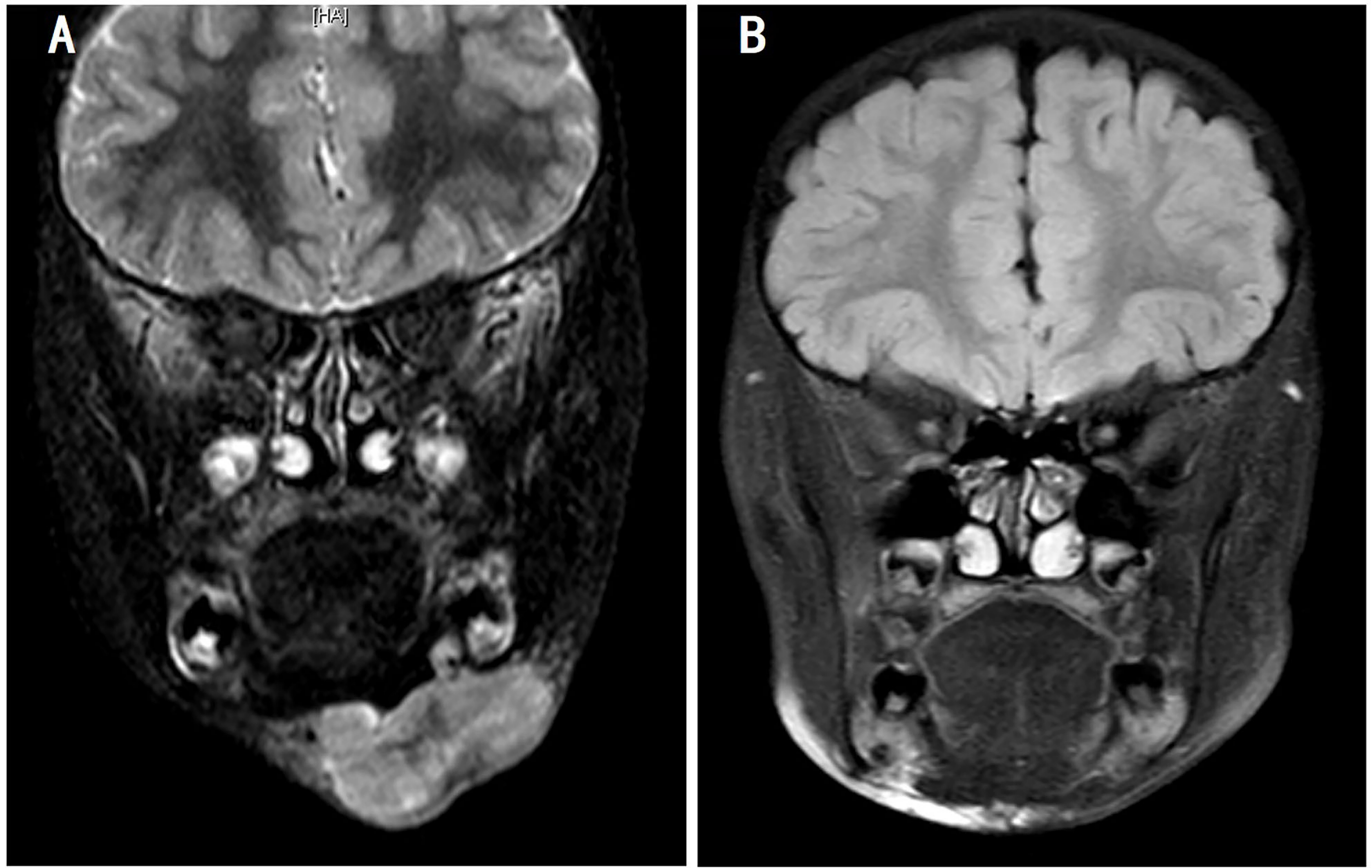
Magnetic resonance imaging of the changes in patient 2. **(A)** Before ATO treatment and **(B)** 3 weeks after three courses of treatment with the ATO-combined chemotherapy (five courses in total). ATO, arsenic trioxide.

Patient 2 was diagnosed with ARMS when he was 2 years old with a single lesion in the left ala nasi. He had received 30 weeks of standard chemotherapy and midterm radiotherapy but exhibited a tumor recurrence in the left mandibular area. The patient once gave up further treatment after a radical operation. After 1 year, when he came back to the hospital, the general imaging examination showed the presence of a mandibular mass of 44 × 38 × 28 mm with multiple lymph node metastases in the bilateral carotid sheath (**Figure 3**). Glioma-associated transcription factor-1 (GLI1) amplification was detected by targeted next-generation sequencing (NGS) in the tumor tissue. When the patient finished the second course of ATO combined with the VAC regimen, a 90% reduction in tumor volume was observed. Unfortunately, he discontinued this novel protocol for ~7 months, and the mass grew up to three times despite trying Chinese herbs. However, once he was treated with ATO-combined chemotherapy again, the tumor shrank rapidly, and no lesion was revealed by MRI after the fifth course (**Figure 4**). Now he has finished nine cycles of the ATO-combined chemotherapy in total for >3 months and has achieved continuous CR.

Patient 3 was a 12-year-old child with ARMS (89 × 6 × 60 mm) on the right forearm. ATO-combined chemotherapy was based on the results of core needle biopsy. As determined by MRI, the tumor volume was reduced by 98% after four courses of the novel regimen. Radical resection and proton radiotherapy were employed successively to eliminate the residual lesion, and four more courses of the ATO-combined chemotherapy were performed to consolidate its curative effect. Presently, the patient remains CR and sticks to the following consolidation therapy.

Patient 4 was a 4-year-old girl who suffered from refractory and recurrent ARMS disease with distant metastases. Then, 2 years ago, she was diagnosed with a primary tumor on the left buttock, which was classed as stage 3 (T2bN0M0). She received first-line comprehensive therapy consisting of VAC and an ifosfamide-and-etoposide regimen but relapsed after radical operation and radiotherapy. Orienting for a possible targeted therapy, several gene amplifications were found by targeted NGS, including PIK3CA, MER proto-oncogene, tyrosine kinase, insulin-like growth factor 2, and GLI2. However, although a variety of second-line drugs, including vinorelbine, paclitaxel, nedaplatin, keytruda, pazopanib, anlotinib, temsirolimus, and everolimus, were tried successively, the primary tumor progressed, developing a distant metastasis in the process. At the time she was treated with the ATO-combined chemotherapy, large masses on the left buttock (66 × 46 × 29 mm) and the right back (33 × 26 × 18 mm), with metastasis to the orbicular mass 36 mm in diameter in the lung region, were observed. After eight cycles of ATO-combined chemotherapy, the tumor volumes of the primary lesion and metastases reduced by 64.7 and 59.2%, respectively. Due to the lack of other effective treatments, three more courses of ATO-combined chemotherapy were added, but the evaluation resulted in stable disease of the tumor. In the follow-up visit, the patient was transferred to another hospital by her own will and has now survived with the disease being stable.

All patients exhibited grades II and III nausea and vomiting during treatment, but this was suitably controlled with antiemetic treatment and nutritional support. Myelosuppression occurred in every patient after each course of ATO-combined chemotherapy, and hematologic recovery was achieved at 2 to 3 weeks later. Grades III and IV neutropenia were the most common form of myelosuppression; two patients therein suffered from febrile neutropenia without focal infection. Chemotherapy-induced thrombocytopenia and anemia were mild with component blood transfusion. Except for these, no other adverse event was observed during the period of treatment.

## Discussion

At present, the treatment of ARMS remains a difficult task in view of its unsatisfactory response to conventional treatment. Therapy is limited due to local tumor recurrence, development of metastases, and multidrug resistance. Patients with progressive or recurrent ARMS have a poorer prognosis, with a 5-year survival of <20% (4, 15). In recent years, researchers have paid increasing attention to targeted treatments since the molecular basis of ARMS is better understood. Activation of the Hh pathway is demonstrated to be responsible for a variety of human cancer types, including RMS. It has been reported that the Hh signaling molecules, such as Sonic Hh (SHH), protein patched homolog (PTCH), Smoothened (SMO), and GLI, are expressed in 70–90% of ARMS patients (6, 7). Targeting on these frequently upregulated molecules may be promising treatments for ARMS. In fact, in *in vitro* experiments, certain SMO inhibitors, such as cyclopamine, GDC-0449, LDE225, and HhA, have been preliminarily shown to exhibit a significant inhibitory effect on tumor growth and the metastasis of ARMS or transplanted tumor models (16). However, there is increasing evidence showing acquired resistance to currently used SMO inhibitors (17). Furthermore, activation of the non-canonical Hh pathway independent of SMO demonstrated that the GLI transcription factors can be activated by other signaling pathways, such as PI3K/AKT/mTOR, TGF-β/SMAD3, RAS/RAF/MEK/MAPK, and KRAS/AR pathways (18, 19). Thus, searching for GLI targeted inhibitors is more crucial for ARMS therapy.

**Figure 5:**
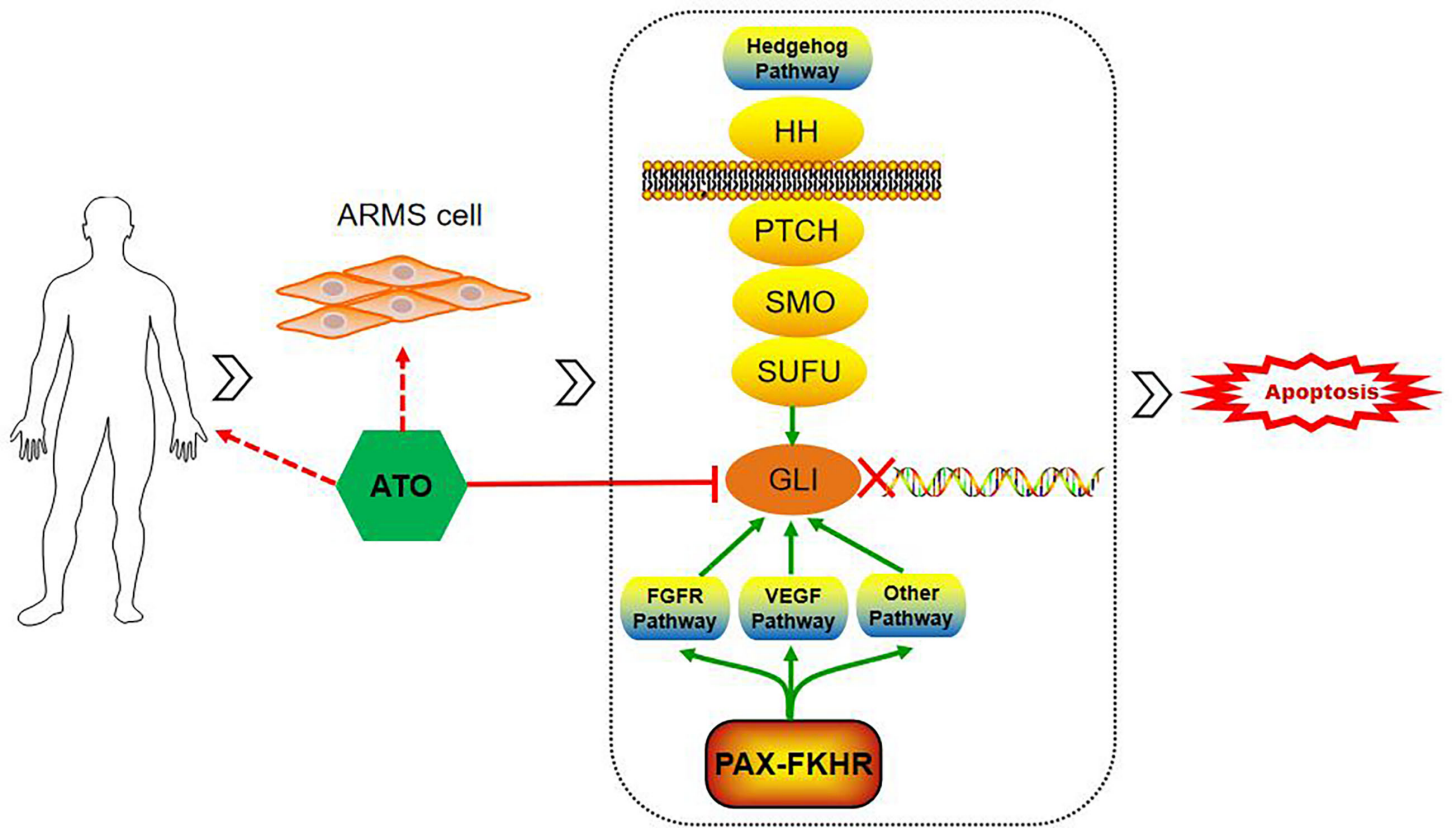
Schematic of ATO inducing apoptosis and inhibiting the tumor growth of alveolar rhabdomyosarcoma by targeting GLI. GLI-dependent tumorigenesis can be stimulated by the activation of the hedgehog pathway and cross-talk with PAX-FOXO1 *via* several pathways, such as FGFR and VEGF pathways. ATO, arsenic trioxide; GLI, glioma-associated transcription factor; PAX-FOXO1, generate paired box-forkhead box O 1; FGFR, fibroblast growth factor receptor; VEGF, vascular endothelial growth factor.

ATO is an old drug approved by the FDA for APL treatment and was later rediscovered as a Hh pathway inhibitor. The Hh pathway is an important signal transduction system for embryonic development, tissue regeneration, and homeostasis maintenance. The main signal axis of the canonical Hh pathway consists of the ligand protein SHH, the transmembrane receptor proteins PTCH and SMO, the suppressor of fused protein SUFU, and the nuclear transcription factor GLI. GLI regulates the expression of downstream genes involved in proliferation, angiogenesis, and stem cell self-renewal (20). Among the current FDA-approved inhibitors of the Hh pathway, ATO is the only agent targeting at the level of the GLI proteins. In 2014, German researchers Michael Meisterernst and Michael C. Frühwald first reported the anti-tumor effects of ATO in malignant rhabdoid tumors *in vitro* and *in vivo* by targeting overexpressed GLI1 (8). Boehme KA et al. verified the selective toxicity of ATO on RMS cell lines, without injury on normal skeletal muscle cells, and ATO was more efficient than the other Hh pathway inhibitors (9). The antagonistic effects of ATO are achieved by ATO binding directly to the GLI zinc fingers, which is similar to the domain of promyelocytic leukemia–retinoic acid receptor α in APL, leading to the transcriptional inactivation of GLI (21). This inhibitory effect of ATO was confirmed in the present study based on the incredibly favorable therapeutic response and outcomes in patients 2 and 4, who assessed the effects of genetic abnormality of GLI1 amplification. Unfortunately, genetic testings for GLI amplifications or mutations were not performed, for personal reasons, in patients 1 and 3. However, they were both found to be PAX3-FOXO1 fusion-positive, which is recognized as an ARMS-specific gene fusion. PAX3-FOXO1 is revealed to contribute to tumorigenesis by activating a series of transcription targets, such as fibroblast growth factor receptor (FGFR), insulin-like growth factor 1 receptor, and vascular endothelial growth factor receptor (VEGFR) (22). Additionally, GLI-dependent tumorigenesis can be stimulated by the cross-talk of FGFR and VEGF pathways (23). Hence, it is hypothesized that ATO exerts its effect on cases of PAX3-FOXO1 fusion-positive ARMS as well (**Figure 5**). Of note is that the functional mechanism of ATO is manifold—for example, it has been shown that ATO can induce apoptosis by increasing the intracellular reactive oxygen species concentrations and the activation of caspase-dependent apoptosis pathways, inhibit tumor-induced angiogenesis by downregulating VEGF, and induce cell cycle arrest in the G2/M phase by regulating cyclinB and Bcl-2 (24). Therefore, it is not surprising that ATO may act synergistically with other agents, such as vincristine, and enhance the chemosensitivity to ARMS (10). As a consequence, the pleiotropic effects of ATO may contribute to the overall response rate in the treatment of ARMS.

Since ATO has been used clinically for several decades, a variety of studies have demonstrated possible ATO-related toxicities. At pharmacological doses of ATO, expected toxicities of acute arsenic exposure include somatic symptoms and organ damage, which usually present in the gastrointestinal, hematological, cardiovascular, and nervous systems (25). When similar doses of ATO were added to the traditional VAC regimen, as observed in our patients, the prominent side effects were gastrointestinal complaints and hematological abnormalities. However, common cardiotoxic effects, such as QT prolongation and ventricular tachycardia, were not observed in the present study. Thus, it may be deduced that the novel regimen of ATO-combined chemotherapy with VAC did not result in increased acute toxicity, though later-onset toxicities have not been determined as of yet.

Our study evaluated the activity and safety of ATO-combined chemotherapy with a VAC regimen in ARMS patients for the first time. As was shown in this pilot clinical trial, all of the four patients, including those with untreated and pretreated ARMS, were sensitive to ATO-combined chemotherapy and achieved partial remission or CR during treatment. It is noteworthy that patient 2 showed a continuous good response to the ATO-combined chemotherapy even though he exhibited repeated relapse. Although patient 1 died of relapse and patient 4 seemed to lose sensitivity to additional combined chemotherapy, these results do show that refractory patients are likely to benefit from ATO-combined chemotherapy with prolonged remission and survival times. Furthermore, ATO-combined chemotherapy with the VAC regimen seemed to cause no additional acute toxicity. From the details mentioned above, it is suggested that ATO, combined with the VAC regimen, exhibits beneficial activity against ARMS in pediatrics. There may be some limitations, such as small sample size, lack of a control group, and potential for selection bias in this study. Therefore, prospective large-scale clinical trials are required to determine the long-term efficacy, optimal course, and late toxicity of ARMS patients.

## Data Availability Statement

The original contributions presented in the study are included in the article/supplementary material. Further inquiries can be directed to the corresponding author.

## Ethics Statement

The studies involving human participants were reviewed and approved by the Ethics Committee of Sun Yat-Sen Memorial Hospital. Written informed consent to participate in this study and consent for publication of any potentially identifiable data or images were provided by the participants’ legal guardian/next of kin

## Author Contributions

YL designed the study and reviewed the manuscript. XP performed the study and wrote the manuscript. XX and HL assisted in performing the experiments and in collecting the materials. CF, PW, CL, and WW supervised the study and revised the manuscript. All authors contributed to the article and approved the submitted version.

## Funding

This work was supported by grant SYS-C-202007 from the Sun Yat-Sen Clinical Research Cultivating Program of Sun Yat-Sen Memorial Hospital.

## Conflict of Interest

The authors declare that the research was conducted in the absence of any commercial or financial relationships that could be construed as a potential conflict of interest.

## Publisher’s Note

All claims expressed in this article are solely those of the authors and do not necessarily represent those of their affiliated organizations, or those of the publisher, the editors and the reviewers. Any product that may be evaluated in this article, or claim that may be made by its manufacturer, is not guaranteed or endorsed by the publisher.

## Abbreviations

ARMS, alveolar rhabdomyosarcoma; ATO, arsenic trioxide; Hh, hedgehog; VAC, vincristine, actinomycin, and cyclophosphamide; VCR, vincristine; PAX-FOXO1, generate paired box-forkhead box O 1; IE, ifosfamide and etoposide; IRS, Intergroup Rhabdomyosarcoma Study group; PR, partial remission; CR, complete remission; PD, progressive disease; SD, stable disease; APL, acute promyelocytic leukemia; PET–CT, positron emission tomography–computed tomography; MRI, magnetic resonance imaging; GLI, glioma-associated transcription factor; FGFR, fibroblast growth factor receptor; VEGFR, vascular endothelial growth factor receptor; NGS, next-generation sequencing.
